# Benefits of Intra-Aortic Balloon Pump Support in Patients with Postcardiotomy Shock Requiring Venoarterial Extracorporeal Membrane Oxygenation

**DOI:** 10.3390/life12081195

**Published:** 2022-08-05

**Authors:** Dejan Radakovic, Kiril Penov, Khaled Hamouda, Nodir Madrahimov, Darko Radakovic, Constanze Bening, Rainer G. Leyh, Ivan Aleksic

**Affiliations:** 1 Department of Thoracic and Cardiovascular Surgery, University Hospital Würzburg, 97080 Würzburg, Germany; 2 Clinic for Thoracic and Cardiovascular Surgery, Heart and Diabetes Center NRW, Ruhr-University Bochum, 32545 Bad Oeynhausen, Germany

**Keywords:** postcardiotomy shock, VA-ECMO, IABP

## Abstract

Background: The benefit of the combined use of an intra-aortic balloon pump (IABP) and venoarterial extracorporeal membrane oxygenation (VA-ECMO) for postcardiotomy shock remains unclear. We aimed to analyse the potential benefits and safety of combining these two devices. Methods: We enrolled 200 patients treated with either VA-ECMO only or in combination with IABP (ECMO-I group) between January 2012 and January 2021. To adjust the patients’ backgrounds, we used propensity score matching for additional analyses, resulting in 57 pairs. The primary endpoint was 30-day survival. Secondary endpoints included successful weaning and complication rates. We also analysed hemodynamic parameters in both groups. Results: After propensity score matching, 30-day survival was better in the ECMO-I group (log-rank *p* = 0.004). The ECMO-I and ECMO-only groups differed regarding the secondary endpoints, including successful weaning (50.9% and 26.3%, respectively; *p* = 0.012) and the need for continuous renal replacement therapy (28.1% and 50.9%, *p* = 0.021). Complication rates were not statistically different between the two groups. Conclusion: Compared to VA-ECMO alone, the combined use of VA-ECMO and IABP is beneficial regarding 30-day survival in selected patients with postcardiotomy shock; successful ECMO weaning and freedom from renal replacement therapy is more common in patients supported with VA-ECMO plus IABP.

## 1. Introduction

Postcardiotomy shock (PCS) after adult cardiac surgery remains a life-threatening complication with an incidence between 0.5–6% and mortality rates reaching 60% [1]. It is caused by severe impairment of myocardial function that results in low cardiac output, end-organ hypoperfusion and hypoxia. In hemodynamically unstable patients unresponsive to medical treatment, venoarterial extracorporeal membrane oxygenation (VA-ECMO) is used for circulatory support and for improvement of gas exchange in cases of severe respiratory failure [2]. It can be established either by cannulation of both femoral vein and artery, or in cases of central cannulation by placing the outflow arterial cannula directly into the aorta [3].

An intra-aortic balloon pump (IABP) is often used as a first step of escalation towards mechanical support, or as an assisting circulatory device. Its safety profile, ease of insertion and efficacy makes its use popular and reasonable. Its primary benefit is a reduction in the myocardial oxygen supply–demand ratio. Further benefits include support of coronary circulation and reduction in left ventricular stress and cardiac work-load [4]. However, in randomised trials, the IABP failed to show a benefit for treatment of cardiogenic shock itself [5]. Nevertheless, it is still routinely used by many cardiac surgery units and some articles report its usage with improved functional outcomes [6].

The combined use of IABP and VA-ECMO for postcardiotomy shock is poorly defined. This is caused by different studies showing disparate outcomes and benefits of concurrent use of the two systems [7,8]. There are no large meta-analyses or randomised trials dealing with this topic in surgical patients with postcardiotomy shock. Contemporary guidelines regarding the treatment of PCS suggest that concomitant application of VA-ECMO and IABP should be considered, but also demand further studies to gather more evidence in this regard [9].

Current concepts of treatment of cardiogenic shock suggest circulatory support, ventricular unloading, and myocardial perfusion as vital treatment aspects in order to improve survival [10]. Since the IABP is the least invasive and cheapest temporary mechanical support option, we wanted to determine if in patients with PCS the combination of IABP and VA-ECMO can improve the outcome without increasing the complication rate.

## 2. Materials and Methods

### 2.1. Study Design and Patients

**Figure 1:**
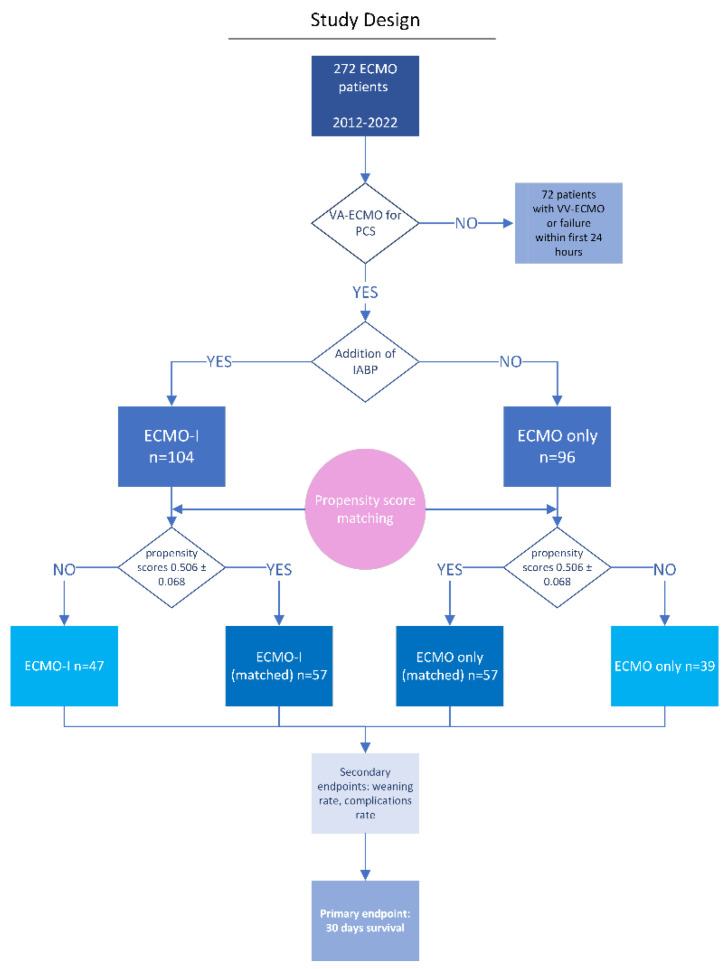
Flow diagram. VA-ECMO—venoarterial extracorporeal membrane oxygenation; VV-ECMO—venovenous extracorporeal membrane oxygenation; PCS—postcardiotomy shock; IABP—intra-aortic balloon pump.

This is an observational retrospective single-centre analysis of 272 patients requiring ECMO support between January 2012 and January 2021 (Figure 1). Cardiac surgery patients who required postoperative venovenous (VV-ECMO) support for respiratory failure or with preoperative VA-ECMO support and those who died on VA-ECMO within the first 24 h were excluded from further analysis. We retrospectively reviewed the remaining 200 patients who received VA-ECMO due to postcardiotomy shock. We divided all patients into two groups, those with combined IABP and VA-ECMO support (ECMO-I group) and those with VA-ECMO only (ECMO-only group). The Ethics Committee of the University of Wurzburg approved this study and individual consent was waived (approval: 20200507 04; 8 June 2020).

The decision for VA-ECMO implantation was made by the surgeon during surgery due to the inability to come off cardiopulmonary bypass (CPB) or by the ICU attending staff immediately postoperatively. The most common indications were failure to wean from cardiopulmonary bypass (CPB), refractory cardiogenic shock (systemic arterial pressure < 90 mmHg or cardiac index < 2.1 L/min/m^2^ with adequate preload) and right-heart failure with or without concomitant left-heart failure despite maximum inotropic support. Implantation of an IABP was performed either before surgery as protection during the initiation of anaesthesia if LVEF was severely reduced (LVEF < 35%) or during the operative procedure as a first step of escalation towards mechanical circulatory support during weaning of the CPB. The decision to place the IABP was at the surgeon’s discretion.

### 2.2. Operative Technique

Insertion of the IABP was performed using the sheathless Seldinger technique via the femoral artery with an 8 Fr catheter. We regularly used either the CS300 intra-aortic balloon pump system or the Cardiosave^®^ hybrid system (Maquet Getinge group, Mahwah, NJ, USA). For the VA-ECMO system, we either used a centrifugal pump console (Cardiohelp, Maquet, Rastatt, Germany) or a RotaFlow centrifugal pump (Maquet, Rastatt, Germany) in conjunction with an oxygenator (HLS Module Advanced, Maquet, Rastatt, Germany) with a separate heat exchanger (Deltastream HC, Medos, Heilbronn, Germany). The return (arterial 19, 21 or 23 Fr) cannulation was performed either peripherally via the axillary or femoral artery with a separate distal leg perfusion cannula or through an 8 mm vascular graft or centrally via the ascending aorta through an 8 mm vascular graft. Venous drainage (venous 23 Fr) was achieved using the peripheral cannula inserted into the femoral vein and advanced into the right atrium under transoesophageal echocardiographic guidance.

### 2.3. Postoperative Care and Outcome Variables

The ECMO circuit was primed with normal saline. Activated clotting time (ACT) was targeted at 160–180 s. Anticoagulation using unfractionated heparin was started 6 h after VA-ECMO was started. Activated partial thromboplastin time (aPTT target: 50–70 s), D-dimers, antithrombin III and fibrinogen were measured every 6 h and adjusted accordingly. The VA-ECMO circuit was checked twice a day by a bedside perfusionist and changed if fibrin depositions or clots accumulated on the oxygenator membrane. The VA-ECMO pump flow was adjusted to maintain mixed venous saturation above 60% and a mean aortic pressure above 60 mmHg. Diuretics were used to maintain the fluid balance. Continuous renal replacement therapy (CRRT) (Fresenius Multifiltrate, Version 5.2, Bad Homburg, Germany) was instituted for hyperkalaemia, fluid overload with oliguria (i.e., urine output < 400 mL/day), elevated blood urea levels (typically > 200 mg/dL) and metabolic acidosis. CRRT was performed through a double-lumen catheter inserted into the axillary, internal jugular or femoral vein. Left ventricular ejection fraction was controlled by echocardiography at least once a day to avoid thrombus formation and to control aortic valve opening. Weaning was initiated under stable hemodynamic conditions after an objective assessment of a chest radiograph, arterial and mixed venous saturations, and transoesophageal echocardiography with good end-organ perfusion. Demographic variables (age, sex, preoperative New York Heart Association class, preoperative ejection fraction, type of cardiac procedure, cannulation site, intraoperative parameters and outcome variables, i.e., intra-hospital mortality, reoperations for bleeding or revisions of cannulation site) were extracted from medical records, perfusion protocols, the hospital database and operative records.

### 2.4. Primary and Secondary Outcomes

The primary endpoint was 30-day survival after mechanical circulatory support initiation. Secondary endpoints included rates of myocardial recovery with VA-ECMO weaning. Data were assessed until discharge. Serum lactate and lactate dehydrogenase (LDH) levels were serially assessed. Haemodynamic stabilisation was assessed by haemodynamic values during the VA-ECMO run. Safety assessments during the clinical course included control for peripheral ischaemic complications requiring intervention during the hospital stay, sepsis and need for CRRT.

### 2.5. Statistical Analysis

We evaluated differences in baseline characteristics between groups using standardised differences (SDs), where an absolute value of  >0.2 was considered to be a substantial difference. We performed propensity score matching (PSM) based on the propensity score derived from multivariable logistic regression to balance the two groups. Each patient in the ECMO-I group was matched to one counterpart in the VA-ECMO-only group, if possible. Propensity scores were calculated using sex, BMI, cannulation strategy, baseline serum lactate levels and D-dimer levels. The concordance obtained with this model was represented by a C statistic of 0.86, indicating a strong ability to differentiate between patients with combined IABP + VA-ECMO support (ECMO-I group) and VA-ECMO only (ECMO-only group). For the patients’ clinical characteristics, continuous variables are presented as mean ± standard deviation and categorical variables as frequencies with relative percentage. The normality of the continuous variables was assessed with the Shapiro–Wilk test. The preoperative and postoperative data were compared using a *t*-test for parametric and Wilcoxon’s signed-rank sum test for non-parametric data. Categorical variables are presented as numbers and percentages and compared using the chi-square or Fisher’s exact test, as appropriate. The Kaplan–Meier method was used to examine survival between the groups within the first 30 days after VA-ECMO implantation, whereas the log-rank test was used to compare the curves. A *p*-value of <0.05 was considered significant. All statistics were performed using SPSS software (IBM SPSS Statistics for Windows, Version 23.0, IBM Corp., Armonk, NY, USA) and EZR (Easy Use of R on R commander (Version 1.54), based on R Version 3.5.0).

## 3. Results

### 3.1. Patient Characteristics

We identified 272 patients between January 2012 and January 2021 at our institution who received VA-ECMO treatment for PCS, as shown in Figure 1. We excluded patients with Impella^®^ or other types of left ventricular unloading, and patients who died within the first 24 h after ECMO implantation. Of the remaining patients, 104 were simultaneously supported with an IABP. Analysing the ECMO-I group separately, 74 (71.2%) patients had IABP implantation the same day as ECMO implantation, 23 (22.1%) patients had the IABP implanted a day or two previously to ECMO, and only 7 (6.7%) patients had an IABP implantation on the next day after ECMO began. Analysing only implantations on the same day, the IABP was always implanted before ECMO, either during anaesthesia initiation or while weaning the patient from CPB as a first step in the mechanical device escalation strategy. The baseline characteristics of the ECMO-only group and ECMO-I group before and after propensity score matching are shown in Table 1. In the ECMO-only group, we noticed higher baseline serum lactate levels (6.69 ± 4.89 mmol/L vs. 9.16 ± 6.79 mmol/L, *p* = 0.007) and higher D-dimer levels (3.15 ± 3.58 µg/L vs. 6.81 ± 7.67 µg/L, *p* = 0.003) before matching. Other baseline and treatment characteristics were comparable between the two groups (Table 1). In the ECMO-I group and ECMO-only group, the propensity score before matching was 0.560 ± 0.099 and 0.508 ± 0.127 (*p* = 0.003), respectively. A one-to-one propensity score matching generated a total of 57 pairs, with propensity scores of 0.506 ± 0.068 and 0.506 ± 0.068 (*p* = 1.000).

### 3.2. Addition of IABP to VA-ECMO and Clinical Outcomes

**Figure 2:**
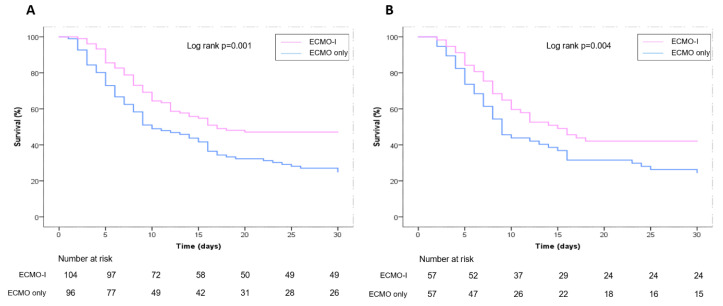
Kaplan–Meier 30-day survival curves. (**A**) Unmatched groups; (**B**) after PS matching.

Overall survival was higher in the ECMO-I group (47.1% vs. 25.0%, *p* = 0.001). The most frequent causes of death during the early ECMO period were due to multiorgan system failure, mesenterial ischemia or sepsis. Survival curves show a better survival in ECMO-I patients compared to the ECMO-only group (Figure 2A). In the ECMO-I group, there was a higher ECMO weaning rate (53.8% vs. 29.2%, *p* = 0.000). Despite better outcomes, patients in the ECMO-I group had a comparable length of stay in the ICU and time on mechanical ventilation (Table 2). The combination of the IABP and VA-ECMO did not cause more peripheral limb ischemia events (6.7% vs. 6.3%, *p* = 0.886).

### 3.3. Addition of IABP to VA-ECMO and Clinical Outcomes in Propensity-Matched Patients

Based on systematically collected data for 27 different variables including baseline demographics and laboratory values, a logistic regression model was used to generate a propensity score for the addition of the IABP to VA-ECMO. Major independent correlates of IABP addition included male sex, body mass index, ECMO cannulation strategy, serum lactate level and D-dimer levels at the time of ECMO implantation.

Baseline characteristics comparing the propensity-matched ECMO-I and ECMO-only patients are shown in Table 1. After propensity score matching, we had 57 pairs with a C statistic of 0.86. As opposed to the entire population, these propensity-matched patients were well matched. Survival curves show better survival within the first 30 days in patients with the IABP plus VA-ECMO compared to the ECMO-only group (*p* = 0.004; log-rank test (Figure 2B)). ECMO weaning was accomplished more often in the ECMO-I group (50.9% vs. 26.3%, *p* = 0.012). Patients supported with VA-ECMO only needed continuous renal replacement therapy more frequently (28.1% vs. 50.9%, *p* = 0.021). There was no difference regarding surgical revisions (24.6% vs. 38.6%, *p* = 0.107) and limb ischemia was rare in both groups (0% vs. 7%, *p* = 0.243).

### 3.4. Hemodynamic Parameters and Laboratory Values

**Figure 3:**
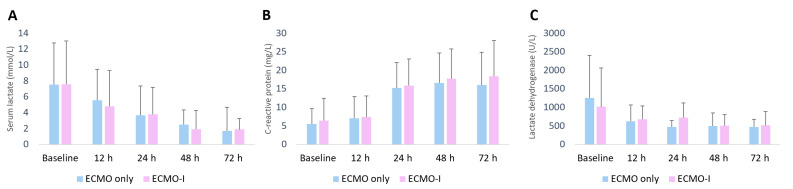
Laboratory values after PS matching. (**A**) Serum lactate levels; (**B**,**C**) reactive protein; (**C**) lactate dehydrogenase.

In both groups after propensity score matching, we saw high initial serum lactate levels but a gradual decrease under mechanical circulatory support and normalisation within the first 48 h (Figure 3A). The same was noted for LDH levels which also decreased over 48 h (Figure 3B). The inflammatory marker C-reactive protein (CRP) increased over the first 72 h under VA-ECMO support but did not differ between groups (Figure 3C).

**Figure 4:**
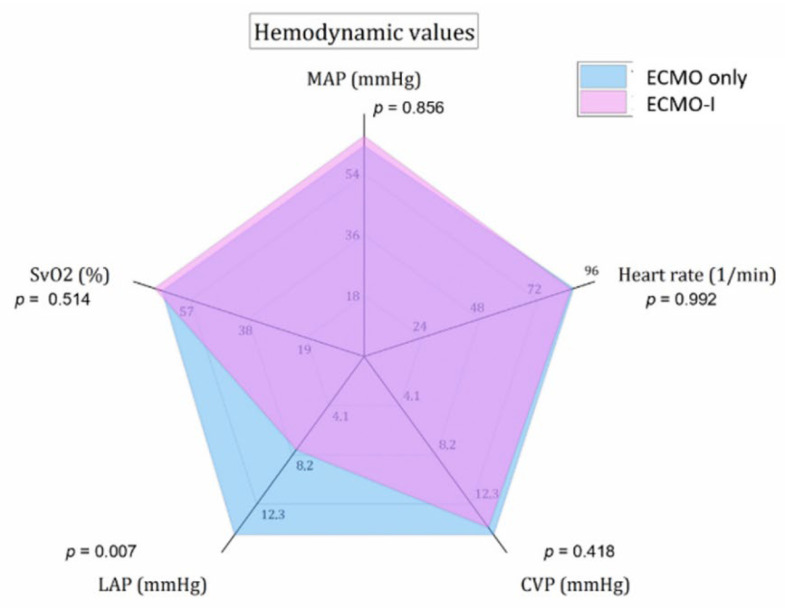
Hemodynamic parameters after PS matching. MAP—mean arterial pressure; CVP—central venous pressure; LAP—left atrial pressure; SvO2—central venous oxygen saturation.

The addition of the IABP to VA-ECMO caused no difference regarding central venous oxygen saturation, mean arterial pressure and central venous pressure over the total period of mechanical circulatory support. Both modalities kept the mean arterial pressure above 60 mmHg. We noticed lower left atrial pressure (LAP) in the ECMO-I group (7.8 ± 3.9 vs. 14.9 ± 6.4 mmHg, *p* = 0.007), as shown in Figure 4. In a subgroup analysis, we noticed the lowest LAP values in patients with central VA-ECMO cannulation combined with IABP support (7.8 ± 3.9 mmHg versus 14.9 ± 6.4 mmHg, *p* = 0.007).

## 4. Discussion

In our study, we found more frequent weaning and better survival within the first 30 days after VA-ECMO implantation in conjunction with an IABP. The lowest LAP values were observed when combining central VA-ECMO cannulation with an IABP. Additionally, in case of peripheral cannulation, the IABP significantly reduced LAP compared to patients without an IABP. LVEDP and mean LAP cannot be used interchangeably, as in early stages of diastolic dysfunction, LVEDP is the only elevated pressure, whereas in settings with increased afterload, mean LAP also increases [11]. Nevertheless, in absence of mitral valve disease, the mean LAP can be a reasonable estimate of LVEDP. Therefore, it is possible that in cases of VA-ECMO patients, the additional IABP utilisation also leads to increased left ventricular compliance, decreased wall tension and a shift of Starling’s law curve to the left as described by other groups in cases of sole IABP therapy [12,13].

In patients with PCS with elevated left atrial pressures, low mixed venous oxygen saturation and other markers of peripheral hypoperfusion despite adequate pharmacological treatment, survival without extracorporeal support is close to zero. In a 15-year analysis of the Extracorporeal Life Support Organization (ELSO) database, ECMO utilisation has increased exponentially for the treatment of PCS over time. However, it appears that survival rates to discharge have decreased during this period [14]. Despite poor results in the IABP-SHOCK II trial, balloon counterpulsation is still widely used as a simple and rapid modality of temporary mechanical support [5].

The concept of combined IABP and VA-ECMO use has already been evaluated by different authors, but their conclusions differ widely. Shotaro A. et al. analysed 1650 patients from the Japanese national database and found improved survival and successful weaning from VA-ECMO if an IABP was added [8]. Kuroki N. et al. reported better neurological outcomes in patients with such combined mechanical support after cardiac arrest [15]. In contrast, Petroni and co-workers demonstrated hemodynamic improvement, but no overall benefit on microcirculation in cases of a combination of an IABP and peripheral VA-ECMO [7]. Despite reduction in pulmonary artery occlusion pressure (PAOP) when combining an IABP with VA-ECMO, PAOP still remained higher due to peripheral VA-ECMO cannulation. Djordjevic et al. found higher weaning rates in VA-ECMO patients after PCS if an additional IABP was used, but did not observe any survival benefit. Only one quarter of their study patients treated with an IABP and VA-ECMO had central ECMO cannulation, and despite no advantage regarding survival, this cohort showed the highest rates of successful ECMO weaning and the lowest mortality rate of all groups screened [16]. Therefore, current recommendations on concomitant use of an IABP and ECMO remain class IIb and level C [9] and call for further investigation of potential benefits.

Acute renal failure can develop in VA-ECMO patients after PCS, which can result in a need for continuous renal replacement therapy (CRRT). CRRT itself also remains an important risk factor for prognosis of VA-ECMO patients. The effects of the IABP upon postoperative renal function were studied by Sloth et al.; they showed that interlobar renal blood flow was higher during counterpulsation [17]. We saw no difference in the need for CRRT in unmatched patients, but a significant decrease in acute renal failure in ECMO-I group patients after PS matching. This could be due to increased arterial pressure during diastole and an increased renal arterial-venous gradient.

We found no difference in complication rates between these two groups. Peripheral vessel puncture did not increase the risk of bleeding or limb ischemia. Our results are comparable to those reported by other groups regarding vascular complications [4].

In case of refractory PCS, VA-ECMO is well established as a rescue measure in the case of LCOS providing better end-organ perfusion. VA-ECMO itself does not necessarily improve failed cardiac function. Due to increased afterload, it shifts Starling’s law curve to the right, increasing the ventricular stroke work even further [18]. With this concept in mind, one would suggest that unloading of the left ventricle plays the most important role for recovery of the failing heart during VA-ECMO therapy. Apart from an IABP, there are different modalities for LV unloading including usage of an axial flow catheter, such as the Impella^®^ device, transapical LV venting, left atrial decompression and transcutaneous or surgical pulmonary artery unloading [19,20]. The combination of VA-ECMO and Impella^®^, first proposed by Pappalardo et al. [21], gained wide interest as it might cause improved outcomes in patients with cardiogenic shock. On the other hand, comparing Impella^®^ and a transapical vent showed no difference in 30-day mortality [20]. Char et al. compared LV unloading for VA-ECMO using an IABP and Impella^®^ and found that only concomitant IABP placement might reduce the morbidity and mortality in VA-ECMO patients for cardiogenic shock [22]. They showed no difference in reducing mean pulmonary pressure by both devices, which is consistent with the theoretical benefits underlying their use.

Several limitations apply to our results: This is a retrospective, single-centre study based on individual physicians’ decisions to add an IABP to VA-ECMO. The decision relied on specific clinical scenarios that might not be readily comparable except for the fact that all patients suffered from postcardiotomy shock, forming a heterogenous group. Nevertheless, we performed a propensity score analysis, controlling for any variables that were significant in the multivariable analysis. After performing 1:1 PS matching, we still found a significant survival benefit in the ECMO-I cohort.

It is important to investigate the right timing for IABP addition to VA-ECMO and identify suitable patients from the PCS collective who will benefit most from IABP, such as patients with low but still preserved LV ejection, suitable heart rhythm and no contraindications for IABP. A prospective randomised trial comparing different LV unloading strategies remains highly desirable for further optimisation of VA-ECMO therapy in patients with PCS.

## 5. Conclusions

Our data indicate better 30-day survival with the addition of an IABP to VA-ECMO for PCS compared to VA-ECMO only. We also found higher weaning rates suggesting better myocardial recovery, less need for renal replacement therapy and no difference in complications when using both instead of only one mechanical circulatory support device.

## Figures and Tables

**Table 1:** Demographic and clinical characteristics of the study population before and after propensity score (PS) matching.

	Unmatched				PS-Matched			
	ECMO-I (N = 104)	ECMO Only (N = 96)	SD	*p* Value	ECMO-I (N = 57)	ECMO Only (N = 57)	SD	*p* Value
Age	65.2 ± 12.13	64.12 ± 12.53	0.08	0.552	65.83 ± 13.55	64.77 ± 11.10	0.09	0.647
Male	78 (75.0%)	63 (65.6%)	0.21	0.146	45 (78.9%)	45 (78.9%)	0.01	1.000
Arterial hypertension	76 (73.1%)	71 (74.0%)	0.02	0.888	41 (71.9%)	37 (64.9%)	0.15	0.546
Body mass index (kg/m^2^)	28.10 ± 4.82	29.39 ± 6.59	0.26	0.117	29.48 ± 7.07	28.54 ± 6.63	0.13	0.431
Smoker	31 (29.8%)	32 (33.3%)	0.15	0.318	15 (26.3%)	19 (33.3%)	0.15	0.539
Diabetes mellitus	28 (26.9%)	27 (28.1%)	0.03	0.849	13 (22.8%)	17 (29.8%)	0.16	0.523
Chronic kidney disease	40 (38.5%)	48 (50.0%)	0.23	0.101	25 (43.9%)	28 (49.1%)	0.11	0.573
Peripheral arterial disease	8 (7.7%)	13 (13.5%)	0.19	0.178	4 (7.0%)	8 (14%)	0.14	0.224
Endocardits	10 (9.6%)	9 (9.4%)	0.01	0.954	7 (12.3%)	3 (5.3%)	0.14	0.321
Cardiopulmonary resuscitation	27 (26.0%)	28 (29.2%)	0.07	0.612	13 (22.8%)	16 (28.1%)	0.12	0.667
SAVE score	−6.14 ± 5.54	−7.02 ± 6.00	0.15	0.487	−5.63 ± 6.32	−6.80 ± 5.52	0.19	0.594
SOFA score	12.75 ± 2.92	13.28 ± 2.72	0.19	0.268	12.72 ± 3.27	12.98 ± 2.74	0.09	0.281
LVEF	33.74 ± 16.86	31.19 ± 16.80	0.15	0.336	31.86 ± 13.52	30.38 ± 13.09	0.11	0.589
TAPSE	17.81 ± 3.53	17.04 ± 5.75	0.16	0.533	16.37 ± 4.20	17.06 ± 6.16	0.13	0.780
Time on CPB	03:11 ± 1:21	3:13 ± 1:09	0.04	0.879	2:53 ± 1:23	3:14 ± 1:14	0.18	0.360
X Clamp time	1:28 ± 0:52	1:31 ± 0:52	0.08	0.755	1:21 ± 0:49	1:26 ± 0:51	0.11	0.583
Operation time	5:20 ± 2:12	4:54 ± 2:05	0.17	0.222	5:01 ± 2:02	4:58 ± 2:01	0.15	0.485
Serum lactate (mmol/L)	6.69 ± 4.89	9.16 ± 6.79	0.42	0.007	7.57 ± 5.44	7.52 ± 5.26	0.01	0.969
C-reactive protein (mg/dL)	7.02 ± 6.72	4.53 ± 5.68	0.18	0.258	6.44 ± 6.04	5.54 ± 4.14	0.18	0.528
Bilirubin (mg/dL)	1.30 ± 1.66	1.53 ± 1.29	0.15	0.47	1.51 ± 2.58	1.69 ± 1.47	0.08	0.759
LDH (U/L)	779.17 ± 954.98	1019.67 ± 1229.21	0.20	0.673	1016.27 ± 1051.96	1250.50 ± 1152.51	0.21	0.259
D-dimers (mg/L)	3.15 ± 3.58	6.81 ± 7.67	0.60	0.003	4.89 ± 4.66	5.63 ± 6.64	0.13	0.313
Albumin (g/L)	1.86 ± 0.53	1.91 ± 0.69	0.10	0.627	1.84 ± 0.45	1.92 ± 0.56	0.16	0.122
ALT (U/L)	345.80 ± 1155.37	279.54 ± 687.72	0.07	0.752	311.84 ± 722.75	384.10 ± 433.09	0.13	0.223
AST (U/L)	161.47 ± 464.81	337.94 ± 802.01	0.19	0.232	341.93 ± 920.41	270.72 ± 719.19	0.09	0.827
Creatinine (mg/dL)	1.51 ± 1.12	1.48 ± 0.80	0.04	0.853	1.49 ± 0.83	1.49 ± 0.92	0.00	0.991
Central cannulation	52 (50%)	38 (39.6%)	0.21	0.139	37 (64.9%)	36 (63.2%)	0.04	1.000

Data are presented as frequencies and percentages (%) or mean ± SD unless specified otherwise. SD—standardised difference = difference in mean or proportions divided by the standard error; imbalance between groups was defined as absolute value greater than 0.20 (corresponding to a small effect size). SAVE—survival after venoarterial ECMO; SOFA—sequential organ failure assessment; LVEF—left ventricular ejection fraction; TAPSE—tricuspid annular plane systolic excursion; CPB—cardiopulmonary bypass; ALT—alanine transaminase; AST—aspartate transaminase.

**Table 2:** Postoperative data.

	Umatched Groups			PS-Matched Groups		
	ECMO-I (N = 104)	ECMO Only (N = 96)	*p* Value	ECMO-I (N = 57)	ECMO Only (N = 57)	*p* Value
Limb ischemia	7 (6.7%)	6 (6.3%)	0.886	0 (0%)	3 (5.3%)	0.243
Surgical revisions	57 (54.8%)	39 (40.6%)	0.242	14 (24.6%)	22 (38.6%)	1.000
Continuous renal replacement therapy	55 (52.9%)	50 (52.1%)	0.332	16 (28.1%)	29 (50.9%)	0.021
Sepsis	38 (36.5%)	34 (35.4%)	0.986	19 (33.3%)	19 (33.3%)	1.000
Time on ECMO (d)	6.5 ± 4.9	6.3 ± 5.4	0.806	5.3 ± 3.3	6.1 ± 5.2	0.509
Lenght of stay (d)	18.9 ± 30.7	15.2 ± 16.3	0.298	13.4 ± 9.1	13.5 ± 14.1	0.956
Weaning	56 (53.8%)	28 (29.2%)	0.000	29 (50.9%)	15 (26.3%)	0.012
Survival	49 (47.1%)	24 (25.0%)	0.001	24 (42.1%)	14 (24.6%)	0.047

ECMO—extracorporeal membrane oxygenation; ICU—intensive care unit.

## Data Availability

The data underlying this article will be shared on reasonable request to the corresponding author.
